# Reactive lymphoid hyperplasia of the thyroid followed by systemic autoimmune diseases: a case report

**DOI:** 10.1186/1752-1947-8-247

**Published:** 2014-07-08

**Authors:** Naoyoshi Onoda, Masahiko Ohsawa, Hidemi Kawajiri, Satoru Noda, Shinichiro Kashiwagi, Tsutomu Takashima, Kosei Hirakawa

**Affiliations:** 1 Department of Surgical Oncology, Osaka City University Graduate School of Medicine, 1-4-3, Ashahi-machi, Abeno-ku, Osaka 545-8585, Japan; 2 Department of Diagnostic Pathology, Osaka City University Graduate School of Medicine, 1-4-3, Ashahi-machi, Abeno-ku, Osaka 545-8585, Japan

**Keywords:** Thyroid tumor, Reactive lymphoid hyperplasia, Thyroid lymphoma, Autoimmune thyroiditis, Pseudolymphoma

## Abstract

**Introduction:**

Reactive lymphoid hyperplasia is a benign nodular lesion characterized by marked proliferation of non-neoplastic, polyclonal lymphocytes forming follicles. The lesion is found in various organs such as skin, orbit, lung, gastrointestinal tract, and liver. However, reactive lymphoid hyperplasia in the thyroid gland is extremely rare. Here, we present an interesting case of reactive lymphoid hyperplasia in the thyroid, which suggests the nature of the disease.

**Case presentation:**

A 74-year-old Japanese man was referred to our institute because of a growing well-demarcated irregular-shaped mass in the right lobe of the thyroid. Malignant lymphoma was suspected by cytology, and right lobectomy was conducted. A final diagnosis of reactive lymphoid hyperplasia was made by the intimate investigation of the surgical specimen, with evidence of polyclonal and non-neoplastic lymphatic proliferations forming follicles with an active germinal center. After an initial uneventful postoperative course, our patient developed severe symptoms of systemic rheumatic arthritis, and alterations in autoimmune reaction, including clinically overt chronic thyroiditis, were identified.

**Conclusions:**

Our case demonstrated important clinical information on reactive lymphoid hyperplasia of the thyroid, and suggested the importance of differential diagnosis, and possible close correlation between systemic autoimmune disorder and the disease.

## Introduction

Reactive lymphoid hyperplasia (RLH) is a term that has been used for localized tumor-forming lymphoid hyperplasia in extranodal organs [1]. The etiology is unknown and it has been called ‘pseudolymphoma’ [2,3] or ‘nodular lymphoid hyperplasia’. RLH is a benign nodular lesion, histopathologically characterized by marked proliferation of non-neoplastic, polyclonal lymphocytes forming follicles with an active germinal center. The lesion is found in various organs such as skin, orbit, lung, gastrointestinal tract, and liver [4]. Although the relationship between RLH and chronic thyroiditis and other autoimmune disease have been discussed [4,5], involvement of this disease in the thyroid is extremely rare. We experienced a case of RHL in the thyroid gland followed by systemic rheumatoid arthritis that became symptomatic soon after surgery. In this report, we will discuss the differential diagnosis of RLH of the thyroid gland, and the possible association with systemic immune disorders.

## Case presentation

A 74-year-old Japanese man underwent an ultrasound examination for the evaluation of cervical atherosclerosis during which an incidental thyroid tumor was discovered. He had had a cerebral infarction eight years previously, which had been treated conservatively without major complication. There were no familial histories of thyroid disease or immune disorder claimed. Fine-needle aspiration cytology (FNA) of the thyroid tumor revealed clusters of many large atypical cells varying in size, and poorly differentiated thyroid carcinoma was suspected. He was, then, referred to our institute for further treatment.

**Figure 1:**
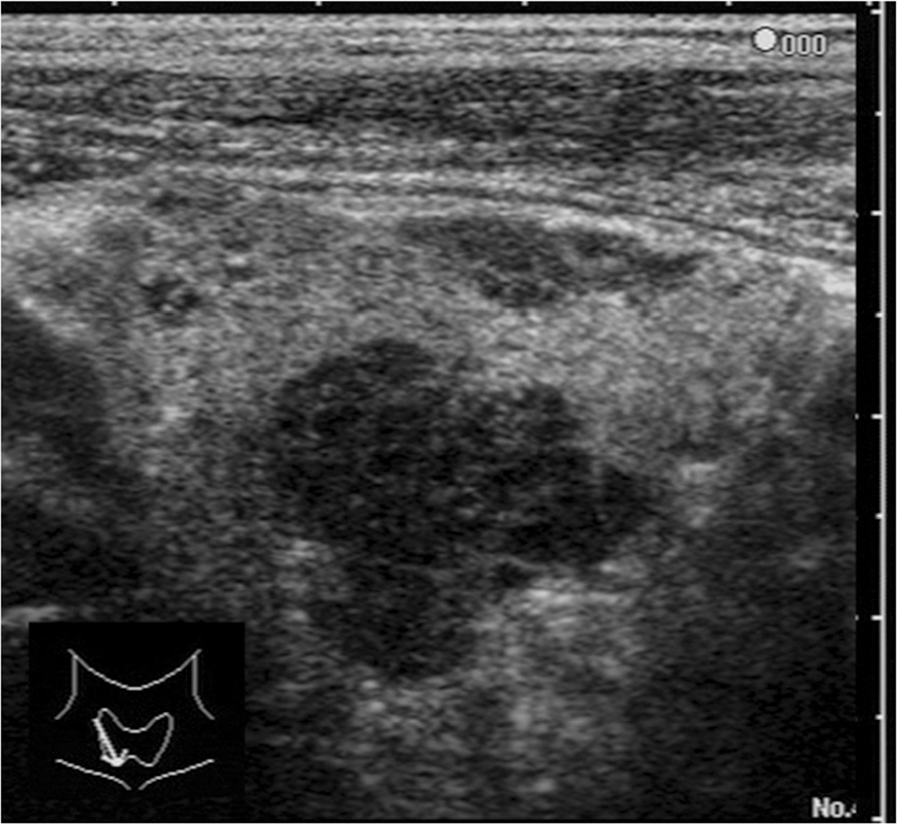
**A representative ultrasonographic image.** A well-demarcated lobular low-echoic lesion with diffuse short linear high-echoic spots inside in the thyroid was revealed by ultrasonography.

On admission, he did not present with rapid enlargement of the tumor, dysphagia, hoarseness, or compression symptoms. The right lobe of the thyroid was diffusely swollen with a smooth surface and normal consistency. No local inflammatory reaction, such as redness and edema on the skin or tenderness, was recognized. No cervical lymphadenopathy was palpable. There were no abnormal findings in his blood count, blood chemistry, and thyroid function. The serum levels of calcitonin, carcinoembrionic antigen, and thyroglobulin were within the normal limits (Table 1). An ultrasonographic examination demonstrated swelling of the right lobe of the thyroid. Several irregular-shaped lesions were found in the right lobe with a maximal diameter of 21mm. Each lesion showed similar appearance; a well-demarcated lobular low-echoic lesion with diffuse short linear high-echoic spots inside (Figure 1). A similar irregular-shaped mass was revealed by contrast-enhanced computed tomography. No abnormal nodule in the mediastinum or the lung was found. Several reactive lymph node swellings were found along with right juglar chain. Repeated FNA revealed normal thyroid follicular cells with abundant lymphatic cells in the background. However, there was no evidence of tumor cells. Right lobectomy was conducted intending to clarify the pathological diagnosis with the suspicions of lymphoma, or poorly differentiated cancer of the thyroid according to the information of the primary physician.

**Table 1:** Laboratory data on admission

WBC	7200/μl	(4300-8000)^*^	BUN	21.0 mg/dl	(7-18)
RBC	487 x10^4^/μl	(450-510)	Creatinine	0.88 mg/dl	(05-1.1)
Hemoglobin	15.1 g/dl	(12.4-17.2)	Sodium^+^	142 mEq/l	(137-146)
Hematocrit	47.1%	(38-54)	Potassium^+^	4.7 mEq/l	(3.8-5.1)
Platelet count	20.4 x10^4^/μl	(18-34)	Chlorine^-^	108 mEq/l	(98-108)
Total protein	7.1 g/dl	(6.5-8.5)	Free T3	3.52 pg/ml	(1.71-3.71)
Albumine	4.2 g/dl	(3.5-5.0)	Free T4	1.21 ng/dl	(0.70-1.48)
AST	21 IU/l	(13-33)	TSH	2.37 μIU/ml	(0.35-4.94)
ALT	15 IU/l	(8-42)	Calcium	9.8 mg/dl	(7.0-10.0)
Total-Bililubin	0.8 mg/dl	(0.2-1.0)	Phosphorus	3.7 mg/dl	(2.9-4.3)
Alkaline phosphatase	210 IU/l	(115-359)			
LDH	177 IU/l	(119-229)	Thyroglobulin	<5.0 ng/ml	(0-30)
Creatine kinase	97 IU/l	(30-200)	Calcitonin	33.0 pg/ml	(5-86)
Glucose	100 mg/dl	(70-105)	CEA	4.2 ng/ml	(0-5.0)
Total-Cholesterol	232 mg/dl	(130-220)	SCC	0.8 ng/ml	(0-1.5)

*Reference range; WBC: white blood cell count; RBC: red blood cell count; AST: asparate aminotransferase; ALT: alanine aminotransferase; LDH: lactate dehydrogenase; BUN: blood urea nitrogen; T3: triiodothyronin; T4: thyroxine; TSH thyroid stimulating hormone; CEA: carcino embryonic antigen; SCC: squamous cell carcinoma antigen.

**Figure 2:**
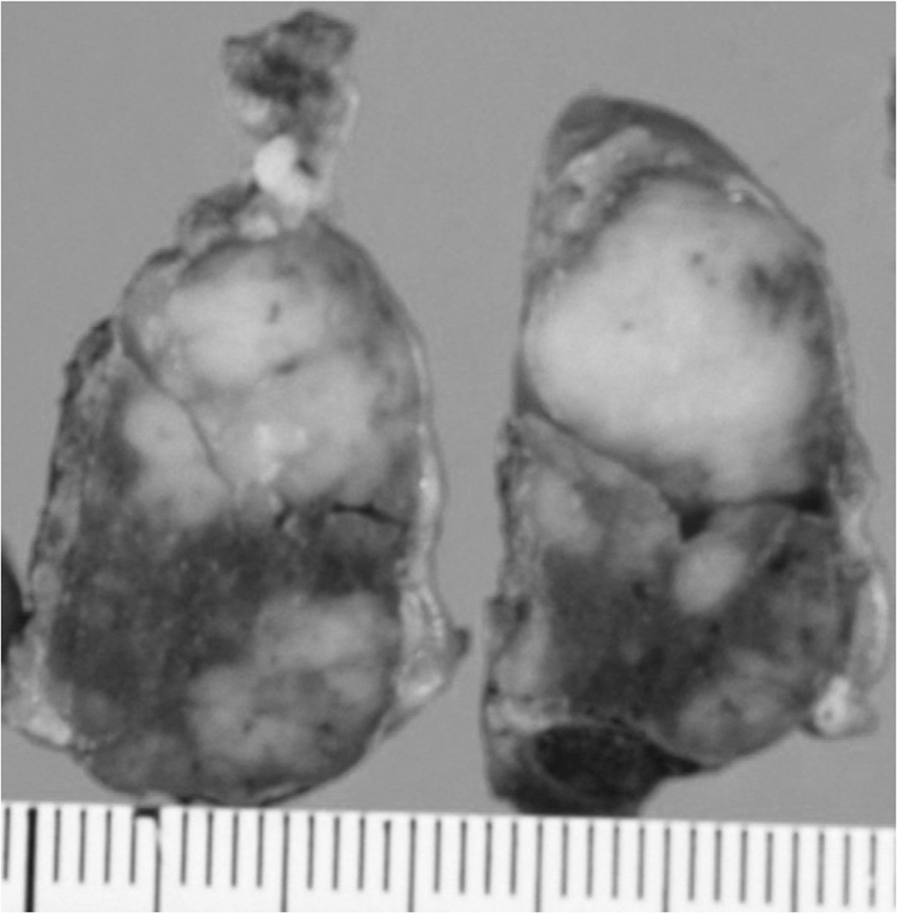
**The macroscopic cross-sectioned view of the surgical specimen.** A well-demarcated, milky-white mass was found in the thyroid.

**Figure 3:**
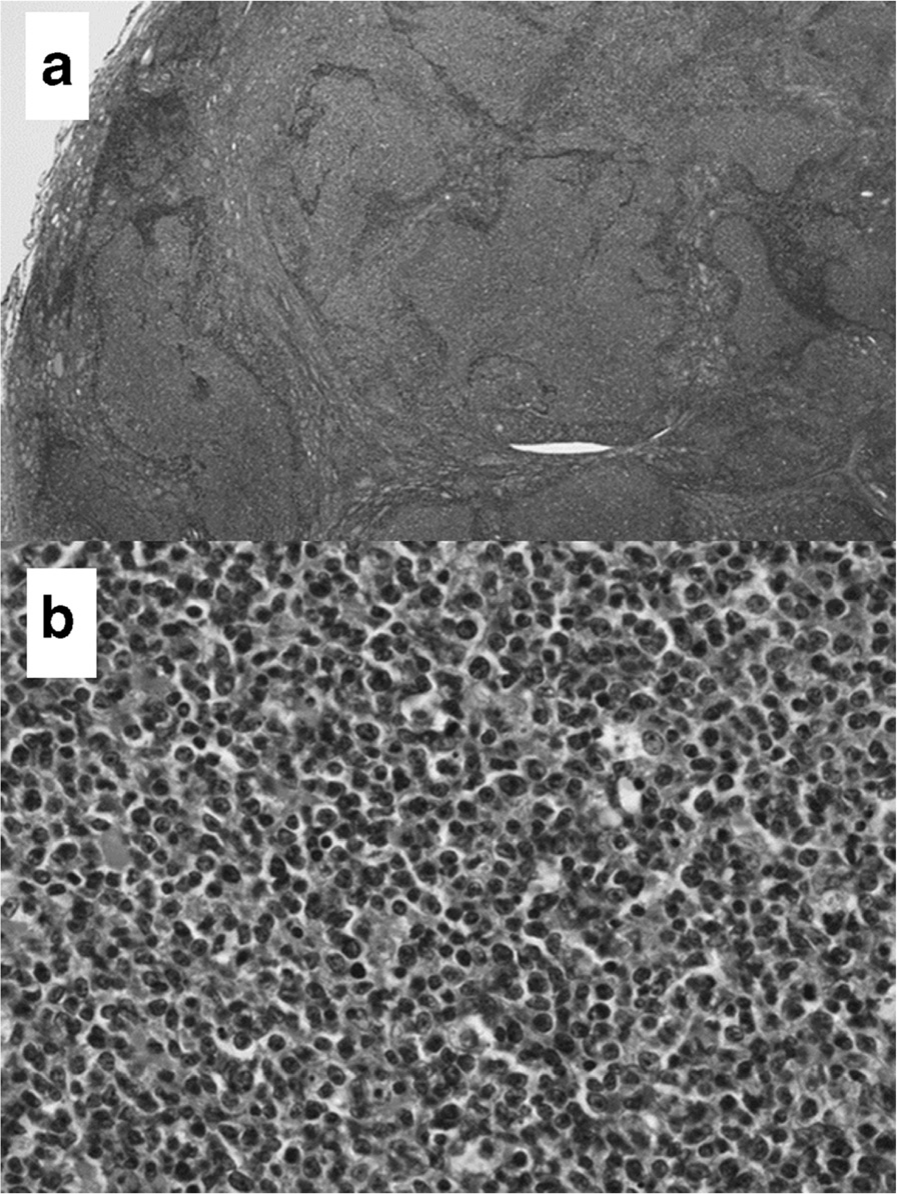
**The microscopic view of the specimen.** Pathologically, marked lymphatic infiltrations were found forming various nodules of lymphatic proliferations (**a**: hematoxylin and eosin stain x40). A mixture of small and large lymphocytes with irregular nuclear borders was shown on high-power field (**b**: hematoxylin and eosin stain x200).

**Figure 4:**
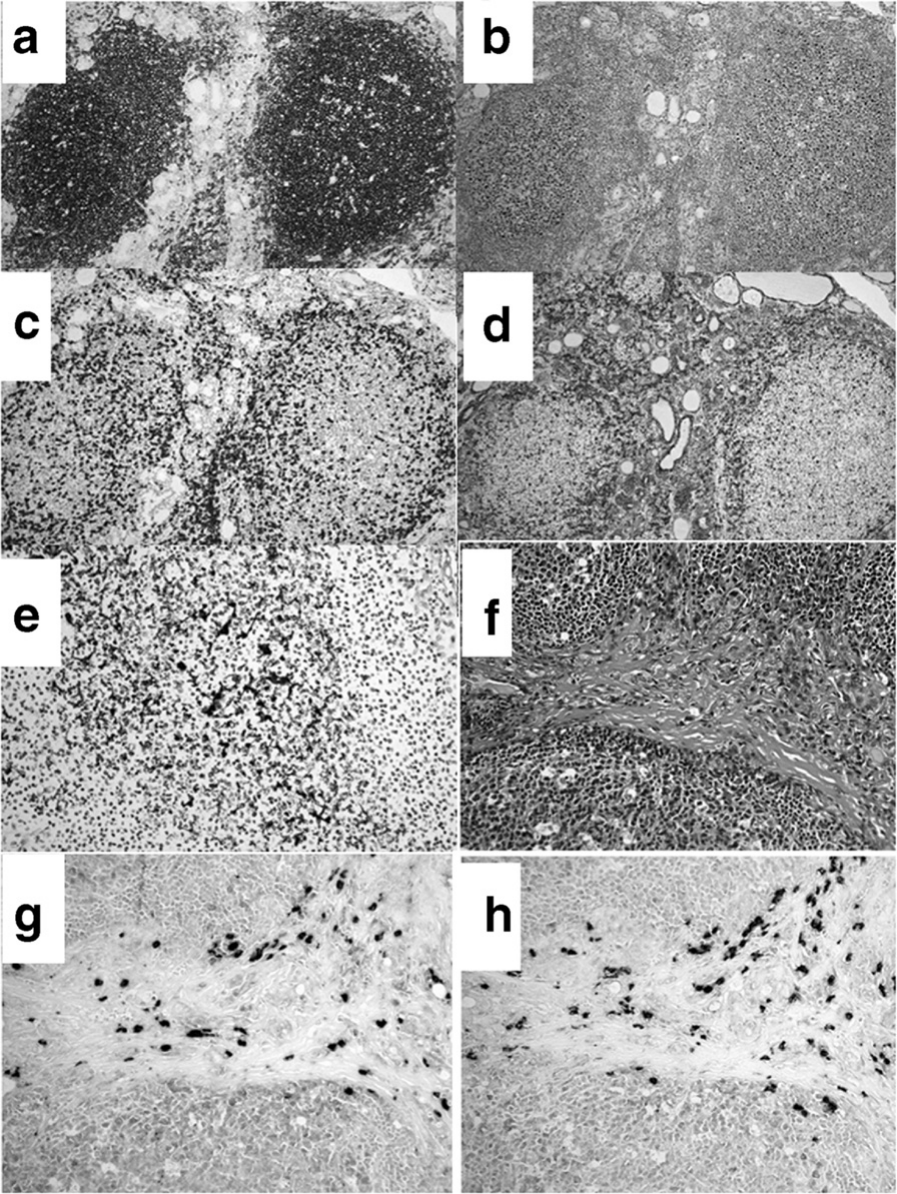
**Representative images of the immunohistochemical study.** B-cell marker (CD-20: **a**), follicular origin B-cell marker (CD-10: **b**), T-cell marker (CD-3: **c**), apoptotic marker (Bcl-2: **d**), follicular-dendric cell marker (CD-35: **e**). Universal reactivity for light chain immunoglobulin were detected by *in situ* hybridization (hematoxylin and eosin stain: **f**, κ: **g,** and λ: **h**).

During the operation, the lesion could not be found on the surface of the thyroid. Several small lymph nodes around the right lobe were recognized. On the cut surface, the tumor was lobular and milky white in color, irregularly but clearly demarcated from the surrounding thyroid gland (Figure 2). A frozen section demonstrated marked lymphoid proliferation suggestive of lymphoma. However, immunostaining for a correct final diagnosis was advised. A pathological investigation of the operative specimen revealed several nodular tumors, leaving normal thyroid tissues between the nodules. Marked lymphatic infiltrations were found within the thyroid gland forming various nodules of lymphatic proliferations. A mixture of small and large lymphocytes with irregular nuclear borders was shown on high-power field. Tingible body macrophage, or mitosis were also recognized. There was no tendency for differentiation to the plasma cell, or no site to show the lymphoepithelial lesion (Figure 3). On immunohistochemical study, both the lymph follicle and the mantle zone were positive for B-cell marker (CD-20: Figure 4a). A diffuse positive nuclear stain of cells within the follicle was shown for follicular origin B-cell marker (bcl-6, CD-10: Figure 4b), but no cells between follicles showed a positive stain for these markers. Various degrees of T-cell marker (CD-3)-positive cell infiltration was found within or around the follicles (Figure 4c). Bcl-2-positive cells were found only at the marginal zone of the follicle. No bcl-2-positive B cell was found in the germ center (Figure 4d). Dendric cells within the follicle were stained positively by follicular-dendric cell marker (CD-35), and demonstrated the preservation of a dense network-like appearance (Figure 4e). Positive reactivity for both κ and λ light chain immunoglobulin (Ig) was found by *in situ* hybridization (Figure 4f, g, h). Furthermore, multiplex polymerase chain reaction (PCR) analysis showed no rearrangement of IgH, indicating polyclonal proliferation of the lymphoid cells (data not shown). A final diagnosis of reactive lymphoid hyperplasia of the thyroid was made according to these pathological findings.

There was no particular event during the initial period of the postoperative course. No abnormal accumulation was found by whole-body Ga schintigraphy. Two months after the operation, our patient began to feel finger stiffness and knee pain. Progressive hypothyroidism became obvious with edema in his lower extremities, and 50μg of levothyroxine was supplemented, but his arthralgia progressed. On additional examination, systemic immune disorders as well as autoimmune reaction to the thyroid gland were demonstrated, and he was revealed to have systemic rheumatic arthritis (Table 2). Two hundred milligrams of bucillamine was initiated and his arthralgia relieved within a month. Postoperative ultrasonography demonstrated diffuse decrease in echogenesity without any tumorous lesion in the residual left lobe of the thyroid gland. Additional impairment of thyroid function was noted. These were consistent with the typical findings of chronic autoimmune thyroiditis. He is well without severe symptoms at 36 months from surgery, with the administration of 100μg of levothyroxine, and 200mg of bucillamine.

**Table 2:** Additional laboratory data*

Free T3	2.23 pg/ml	(1.71-3.71)^**^	Rheumatoid factor	167 IU/ml	(0-20)
Free T4	1.43 ng/dl	(0.70-1.48)	RAPA	640 fold	(<40)
TSH	10.1 μIU/ml	(0.35-4.94)	CH50	40.4 CH50/ml	(28-45)
CRP	2.39 mg/dl	(0-0.4)	Anti-Tg antibody	29.5 U/ml	(<0.3)
Thyroid test^#^	6400 fold	(<100)	Anti-nuclear antibody	negative	(-)
Microsome test^##^	25,600 fold	(<100)	LE test^$^	negative	(-)
Thyroglobulin	5.3 ng/ml	(0-30)			

*Data taken three months after the operation with the supplementation of levothyroxine (50μg); **Reference range; ^#^semi-quantitative test for thyroglobulin; ^##^semi-quantitative test for anti-thyroid microsomal antibody; ^$^agglutination reaction to detect anti-dinitrophenol (DNP) antibody; T3: triiodothyronin; T4: thyroxine; TSH thyroid stimulating hormone; CRP: C-reactive protein; RAPA: rheumatoid arthritis particle agglutination; CH50: 50% hemolytic complement activity.

## Discussion

Saltzstein has defined RLH as propagation of lymph follicles constructed with lymphoid cells without cytological atypia, accompanied with a conspicuous, reactive germinal center [6]. Katayanagi also proposed RLH as a localized lesion well-demarcated from the surrounding tissues and characterized by the presence of hyperplastic lymphoid follicles with polymorphic and polyclonal cell populations composed of small mature lymphocytes, mature plasma cells, macropages and stomal fibrosis [7]. Our case was consistent with these descriptions. As described previously, reports of cases with RLH of the thyroid are extremely rare [2,3]. Clinical and pathological manifestations of thyroid disease with lymphoid infiltrates, such as malignant lymphoma, Graves’ disease, Hashimoto’s thyroiditis, or de Quervain’s subacute thyroiditis, have been well established. Still, typical clinical features of RLH of the thyroid gland have not been described yet. Thus, the differential diagnoses clinically with thyroid tumor or goiter, and pathologically with malignant lymphoma are mandatory.

Franssila [1] clearly demonstrated several important points for accurate differential diagnosis. Our case had no clinical features of hyperthyroidism suggesting Graves’ disease, or no inflammatory reaction suggesting acute to subacute thyroiditis. At the time of surgery, there was no symptom suggesting hypothyroidism. The thyroid nodule was found only in the right lobe, and diffuse change of the thyroid gland was not recognized. These findings indicated the absence of typical autoimmune thyroid disease.

Primary lymphomas represent 1 to 5 percent of thyroid tumors. Most cases occur in patients who have chronic thyroiditis, and there is a distinct female predominance [8]. Pathologically, most thyroid lymphomas are of non-Hodgkin’s B-cell origin [9]. In the present case, differential diagnosis from malignant lymphoma was critical. However, follicular malignant lymphoma should be excluded because of the morphology of the cells, presence of mitosis, or tingible body macrophage. Moreover, the immunohistochemical features, such as normally located bcl-2-positive cells only at the marginal zone, or preserved dense dendric cell network clearly demonstrated the non-tumoral nature of the lymphoid infiltration, and was enough to rule out malignant lymphoma.

Mucosa-associated lymphoid tissue (MALT) lymphoma accounts for 28 to 69 percent of the cases of primary thyroid lymphomas [9,10]. Typical pathological features of MALT lymphoma, such as diffuse monoclonal cell infiltration of centrocyte-like cell or monocytoid B-cell around the germ center, and lymphoepithelial lesions were lacking in the present case. Moreover, both κ and λ light chain immunoglobulin was found, and no rearrangement of IgH was demonstrated, indicating polyclonal proliferation of the lymphoid cells in this case. Therefore, this case did not demonstrate the pathological features compatible with MALT lymphoma.

Focal lymphocytic thyroiditis is a common disorder [11,12]. Focal nodules of thyroiditis have a wide variety of appearance. They most commonly appear as solid hyperechoic nodules with ill-defined margins. However, there are no sonographic features that can reliably diagnose these lesions as thyroiditis and differentiate them from other lesions [13]. Our case demonstrated well-bordered focal hypoechoic nodules, and was, therefore, considered as a tumor rather than a focal lymphatic infiltration at the time of the operation. On the other hand, we could find lymphatic infiltration outside of the principal lesion, not only pathologically in the operative specimens but also clinically after the operation. These findings were suggestive of focal to diffuse lymphocytic thyroiditis in the thyroid gland, and were not indicative for lymphoma. The term focal thyroiditis generally suggested a small reversible lesion accompanied with other main pathological changes. Therefore, we concluded the lesion in the present case was a RLH of the thyroid.

Most RLH is operated on because of the suspicion of malignant disease, and the prognosis is reportedly good [4]. Our case also showed fair prognosis without recurrent disease in the thyroid. However, he developed systemic rheumatoid arthritis, and demonstrated clinically overt progressive autoimmune thyroiditis soon after the operation. The involvement of systemic autoimmune disorder in the RLH [4,5], as well as autoimmune thyroid disease [14], have been reported. Our case had not demonstrated any symptoms indicating systemic immune disorder before initial presentation, although he had been closely followed up for eight years by a local physician after suffering a cerebral infarction. We could, thus, suspect that RLH of the thyroid in this case was initiated as an abnormal local immune reaction caused by a recently appeared primary systemic autoimmune disease.

We should have considered the possibility of focal lymphoid reaction of autoimmune thyroid disease at the moment of initial presentation. However, lack of the typical symptoms and data to indicate chronic thyroiditis, the relatively acute onset of the disease, and the cytological suspicion of the malignant disease lead us to conduct a surgical approach in the present case. Although there remained many points to clarify, our case demonstrated important clinical information of RLH of the thyroid, and suggested possible correlation between systemic autoimmune disorder and the disease.

## Conclusion

We reported a case of reactive lymphoid hyperplasia of the thyroid. Our case demonstrated important clinical information on this rare disease, and suggested the importance of differential diagnosis, and possible close correlation between systemic autoimmune disorder and the disease.

## Patient’s perspective

None given.

## Consent

Written informed consent was obtained from the patient for publication of this case report and any accompanying images. A copy of the written consent is available for review by the Editor-in-Chief of this journal.

## Abbreviations

FNA: fine-needle aspiration cytology; MALT: mucosa-associated lymphoid tissue; RLH: reactive lymphoid hyperplasia.

## Competing interests

The authors declare that they have no competing interests.

## Authors’ contributions

NO performed the surgery and was a major contributor in writing the manuscript. HK, SN, SK, TT, and KH treated the patient, and interpreted the patient data. MO performed the histological examination. All authors read and approved the final manuscript.
